# Melanoma subtypes: genomic profiles, prognostic molecular markers and therapeutic possibilities

**DOI:** 10.1002/path.5213

**Published:** 2019-02-15

**Authors:** Roy Rabbie, Peter Ferguson, Christian Molina‐Aguilar, David J Adams, Carla D Robles‐Espinoza

**Affiliations:** ^1^ Experimental Cancer Genetics The Wellcome Sanger Institute Hinxton UK; ^2^ Cambridge Cancer Centre Cambridge University Hospitals NHS Foundation Trust Cambridge UK; ^3^ Tissue Pathology and Diagnostic Oncology, Royal Prince Alfred Hospital Sydney Australia; ^4^ Melanoma Institute Australia, The University of Sydney Sydney Australia; ^5^ Laboratorio Internacional de Investigación sobre el Genoma Humano Universidad Nacional Autónoma de México Santiago de Querétaro Mexico

**Keywords:** melanoma, cutaneous, desmoplastic, acral, uveal, mucosal, mutations, driver genes, biomarkers, prognostic, predictive, 31‐gene expression profile

## Abstract

Melanoma is characterised by its ability to metastasise at early stages of tumour development. Current clinico‐pathologic staging based on the American Joint Committee on Cancer criteria is used to guide surveillance and management in early‐stage disease, but its ability to predict clinical outcome has limitations. Herein we review the genomics of melanoma subtypes including cutaneous, acral, uveal and mucosal, with a focus on the prognostic and predictive significance of key molecular aberrations. © 2018 The Authors. *The Journal of Pathology* published by John Wiley & Sons Ltd on behalf of Pathological Society of Great Britain and Ireland.

## Introduction

**Figure 1:**
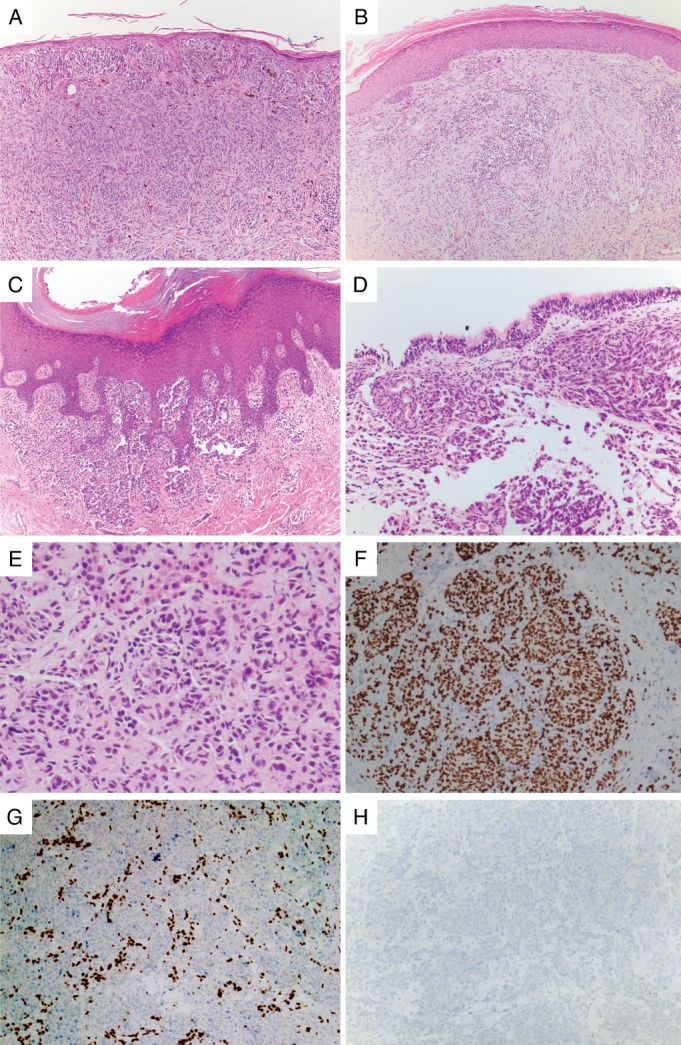
Histopathology of melanoma subtypes. (A) CM of superficial spreading type features an *in situ* component within the epidermis with underlying dermal invasion. (B) Desmoplastic melanoma, a type of CM, is comprised of a dermal proliferation of atypical spindled cells associated with lymphoid aggregates. (C) Acral melanoma often shows a lentiginous (linear) *in situ* growth pattern along the epidermal ridges with underlying invasion into the dermis. (D) Mucosal melanoma arises in non‐keratinising wet mucosa, shown here invading the subepithelial stroma of respiratory type mucosa in the nasal sinuses. (E) Uveal melanoma preferentially metastasises to the liver as pictured here with accompanying immunohistochemistry showing (F) staining for SOX10 in the melanoma cells, (G) loss of BAP1 staining in the melanoma cells with retention of normal staining in hepatocytes and lymphocytes, and (H) no staining for *BRAF* VE1, indicating the absence of a *BRAF* V600E mutation.

Historically, melanoma has been classified into subtypes based on the tissue from which the primary tumour arises. The major such subtypes are cutaneous melanoma (CM), which arises in non‐glabrous skin; acral melanoma (AM), a distinct form that originates in glabrous skin of the palms, soles and nail beds; mucosal melanoma (MM), the rarest subtype, which arises from melanocytes in the mucosal lining of internal tissues; and uveal melanoma (UM) which develops from melanocytes in the uveal tract of the eye (Figure 1). These subtypes have well recognised epidemiological, clinical and histopathological characteristics, and recent studies have described the molecular alterations that underpin some of these attributes. Site of origin seems to correlate best with tumoural somatic profile, with melanomas arising from chronically sun damaged (CSD) sites having a higher mutational burden than tumours arising from non‐CSD sites 1 – a direct consequence of the UV‐induced C>T transitions at dipyrimidines that dominate the majority of CM genomes 2, 3, 4.

Based solely on the occurrence of driver mutations, melanomas have further been classified into four genomic subtypes: *BRAF*‐mutant, *NRAS*‐mutant, *NF1*‐loss and triple wild‐type (TWT) 3, 4. These subtypes do not have distinguishing histopathological features or sites of origin, although there are notable trends; for example, nearly all UMs and the majority of AMs and MMs fall into the TWT category 3. The *BRAF*, *NRAS* and *NF1* driver alterations all activate the mitogen‐activated protein kinase (MAPK) pathway and generally occur at the earlier stages of tumour evolution 5. In CM, it has been proposed that subsequent mutations occur in the *TERT* promoter and in regulators of the cell cycle such as *CDKN2A*, which precede mutations in chromatin remodelers such as members of the SWI/SNF complex and *TP53*, the latter being associated with more advanced stages of primary tumour progression 5. Whether some cells are inherently able to metastasise or whether further genomic alterations are necessary to gain this ability, remains under active investigation 6, 7.

Herein, we describe how melanoma subtypes are shaped by their genomic profiles, and outline our current understanding of prognostic and predictive molecular markers.

## Melanoma subtypes

### CM: dominated by ultraviolet‐induced mutations

#### The genomic landscape of CM

CM generally affects people of European descent and is the commonest reported melanoma subtype. Consequently, the majority of genomic and transcriptomic studies have been performed on CM cases. CM has the highest burden of somatic mutations across the major cancer subtypes, with a mutational landscape that is dominated by the UV mutational signature, primarily C>T transitions as described earlier 2, 3, 4. About 45–50% of CM are *BRAF*‐mutant (principally through mutations at the V600 codon), ∼30% are *RAS*‐mutant (either *NRAS*, principally at codon *Q61*, *KRAS* or *HRAS*), 10–15% are *NF1*‐mutant and about 5–10% are TWT 3, 4 (Table 1). These genomic subtypes differ in their characteristics and clinical presentation. Melanomas that arise on skin with intermittent sun exposure are generally more likely to have a *BRAF* mutation compared with melanomas occurring on chronically sun‐exposed skin 8. Melanomas with *BRAF* mutations are also more common in younger patients, in the superficial spreading histopathologic subtype and on the trunk 9, 10. *NRAS* mutations appear more frequently in older patients, in the nodular histopathologic subtype and on skin with chronic UV‐damaged skin 11, 12. Additional recurrent mutations identified in large‐scale sequencing studies include disruptive variants in *CDKN2A*, *TP53*, *ARID2* and *PTEN*, and 5′ UTR hotspot mutations in *RPS27* and *MRPS31*, both ribosomal proteins 3, 4. Driver alterations and mutational burden are also related; tumours driven by *BRAF* ^V600E^ mutations tend to have fewer somatic mutations than tumours bearing other, possibly less potent, alterations such as loss of *NF1* and activation of *NRAS*, *KIT* and *BRAF* non‐V600E 1. This may be due to these cancers being promoted by additional mutations spread through different biological pathways, and accordingly, tend to present in later life 1. A more recent study has used this information to propose a sequential order in which signalling pathways become disrupted as precursor lesions evolve to invasive melanoma and subsequent metastases 5, 13. More than 50% of advanced CMs have mutations in the *TERT* (telomerase reverse transcriptase) promoter that create binding sites for the E26 transformation‐specific (ETS) family of transcription factors 14. These promoter variants have been shown to be associated with decreased telomere length and poorer survival 15, 16, 17.

**Table 1:** Overview of genomic profile of melanoma subtypes

Biological pathways	Genes	CM	DM‐subtype	AM	UM	MM
**MAPK genomicsubtypes**	∼Total%mut*	**∼90‐95%**	**∼73%**	**∼50‐60%**	**∼100%**	**∼50‐60%**
	*BRAF*	∼ 45‐50% 3, 4	∼0‐5% 13, 43	∼ 10‐35% 3, 44, 45, 46, 47	rarely seen 48, 49	∼0‐21% 3, 45, 50, 51
	*RAS* (mainly*NRAS*)	∼ 30% 3, 4	∼0‐6% 13, 43	∼ 8‐22% 3, 44, 45, 46, 47	rarely seen 48, 49	∼5‐25% 3, 45, 51
	*NF1*	∼10‐15% 3, 4	∼52‐93% 13, 43	∼11‐23% 3, 44, 47	rarely seen 48, 49	∼0‐18% 3, 51
	TWT	∼5‐10% 3, 4	∼7‐48% 13, 43	∼45‐58% 3, 44	∼100% 48, 49	∼65‐75% 3, 51
	*KIT* (mut orgain)	∼5‐10% 3, 4	rarely seen 13, 43	∼3‐36% 44, 46, 47, 52	∼11% 53	∼7‐25% 3, 51, 54
	*GNAQ*	∼1.5‐2.1% 3, 55	rarely seen 13, 43	∼0‐17% 3, 47	∼43‐57% 48, 49 56, 57	∼1‐12% 3, 51
	*GNA11*	rarely seen 3, 55	rarely seen 13, 43	rarely seen 3	∼41‐49% 48, 49 56	∼1% 51
	*MAP2K1 & 2*	∼4% 3	∼7% 13	∼8% 3	∼9% 48	∼0‐11% 3, 50
**Cell Cycle**	∼Total%mut*	∼57% 3	∼70‐75%	∼90%	∼85%	∼36‐75%
	*CDKN2A*(mut)	∼13‐40% 3, 4	∼20‐29% 13, 58	∼0‐3% 3, 44	rarely seen, methylated in ∼50% 59	rarely seen 3, 50, 51, 54
	*CDKN2A*(loss)	∼45% 3	∼18% 13	∼35% 44	∼12% 48	∼10‐38% 3, 50
	*CDK4* (mutor gain)	∼5‐6% 3, 4	∼5% 13	∼9% 3, 44	∼3% 48	∼5‐25% 3, 50
	*RB1*	∼4‐15% 3, 4	∼15% 13	∼9‐17% 3, 44	∼3% 48	∼0‐21% 3, 50
	*TP53*	∼15‐18% 3, 4	∼40‐60% 13, 43, 58	∼6‐54% 3, 44	∼9% 48	∼7‐15% 3, 50
	*CCND1*	∼5‐13% 3, 4	∼2% 13	∼6‐54% 3, 44	∼6% 48	∼25% 3
	*BAP1* (mut orloss)	rarely seen 3	rarely seen 13	rarely seen 3	∼70‐83% (but the great majority of metastatic UM) 48, 49	rarely seen 3, 50, 51, 54
**PI3K/AKT**	*PTEN* (mut orloss)	∼8.5‐40% 3, 4	rarely seen 13	∼26‐28% 3, 44	∼6‐11%, up to 76% with LOH 48, 60	4‐25% 3, 50, 51, 54
**Number ofmutations**		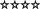	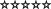	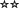	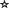	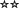
**Chromosomalaberrations**		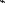	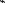	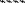	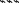	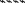
**Transcriptionfactors**	*NFKBIE*promoter	∼5% 3	∼15‐33% 3, 13	not seen 3	NA	rarely seen 3
	*MITF*	∼10‐20% 3, 18	rarely seen 13	∼15% 3	∼63% samples are reported to include deletions or amplifications in MITF 48	∼5‐25% 3, 51
**Telomerasepathway**	*TERT* (mut orgain)	∼85% 3	∼85% 13	∼9‐45% 3, 44, 46	∼2‐9% 48, 61	∼5‐13% 3, 50, 51

* Estimates based on the literature, and on the genes listed on the table including mutations and copy number aberrations.

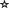
Represents the mutational load.

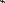
Represents the number of chromosomal aberrations.

The number of individual symbols within each category is proportionate to the number of mutations/chromosomal aberrations.

**Figure 2:**
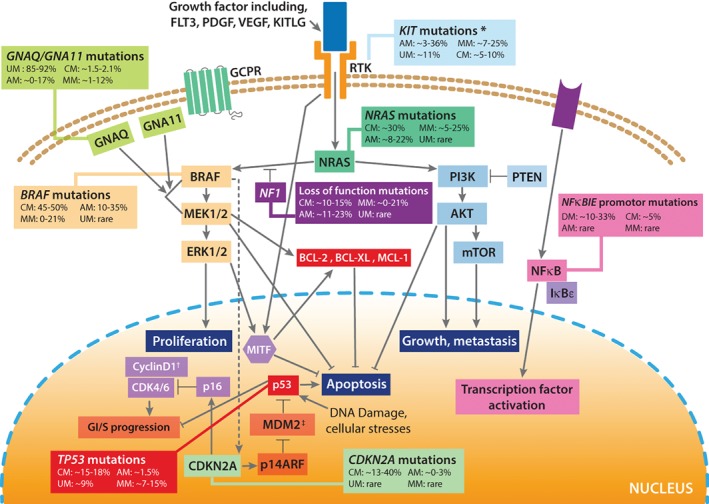
Molecular representation of the mutations associated with the RAS/RAF/MEK/ERK pathways in melanoma, including the MITF signalling cascade. GPCR, G‐protein coupled receptor; RTK, receptor tyrosine kinase. *KIT amplifications are seen in ∼10% of CMs, ∼9.5% of AMs, ∼15% MMs 64. ^†^Cyclin D1 is also amplified in ∼18% of CMs 65. ^‡^MDM2 is also amplified in ∼6% of CMs 66. Adapted from 67.

Microphthalmia‐associated transcription factor (*MITF*) is a melanocyte‐specific transcription factor that binds to the promoter site of multiple target genes involved in melanocyte cell development, pigmentation and neoplasia (Figure 2). *MITF* amplification is present in about 10% of primary melanomas, with a higher incidence reported among metastatic melanomas 18. The role of *MITF* in melanoma progression and resistance to targeted therapy appears paradoxical; some studies have found that CMs expressing *MITF* are well differentiated and have a favourable prognosis 19 and those with low *MITF* expression have an invasive phenotype and are intrinsically resistant to MAPK inhibition 20, whereas others have found that activation of a robust MITF transcriptional program triggers differentiation into highly pigment‐producing drug resistant cells 21. Recent studies have found great heterogeneity in *MITF* expression within tumours 22. An overview of other melanoma pathways and genes is shown in Table 1 and Figure 2.

The relationship between tumour driver mutation status and survival has been the subject of significant research efforts and it is now well appreciated that *BRAF*‐mutant tumours confer a poorer prognosis relative to *BRAF* wild‐type melanoma. In particular, *BRAF*‐mutated melanoma has been linked to a shorter overall survival in patients with stage IV disease when compared to those with *BRAF* WT disease 9, 23. While the majority of studies investigating the relationship between *BRAF* mutations and clinical outcomes are focused on patients with metastatic disease, recent studies have demonstrated that *BRAF*‐mutant melanomas are also associated with a shorter disease‐free and melanoma‐specific survival in patients with early‐stage disease 24, 25. Historically, *NRAS*‐mutant disease has been associated with thicker primary lesions and higher mitotic activity 12. However, there have been conflicting reports on its prognostic significance. In particular, no impact on survival was seen when *NRAS* mutations were measured in primary disease 26, 27, however when measured from metastases, *NRAS* mutations were associated with improved survival compared to tumours with *BRAF* mutations or TWT tumours 28, 29. Despite the undoubted prognostic relevance of American Joint Committee on Cancer (AJCC) classification and certain driver mutations, our ability to predict those early‐stage patients at highest metastatic risk remains conspicuously limited.

#### Gene expression profiles and their prognostic implication

A gene expression profile (31‐GEP) test has been proposed that evaluates the expression of 31 gene targets in the primary tumour, providing a binary classification of ‘low risk’ (Class 1) or ‘high risk’ (Class 2) of metastases within 5 years of diagnosis 30. The test assesses the expression of three control genes, four genes with proven prognostic utility for UMs 31 and 24 genes previously reported to be differentially expressed in metastatic compared to primary tumours 32, 33, 34, 35, 36, 37, 38. The performance of this test has been evaluated in several retrospective 30, 39, 40 as well as prospective validation studies 41, 42 and has been shown to enhance current prognostic accuracy in particular through identifying clinically and pathologically sentinel lymph node (SLN)‐negative patients with high‐risk of metastases. However, although there is great promise in reproducibility and clinical validity, the clinical utility for the 31‐GEP test on clinical decision‐making is still incompletely defined, and will require evidence from further large‐scale prospective multi‐institutional registry studies before it can be considered for inclusion in any national or professional association guideline recommendations.

By undertaking unsupervised hierarchical clustering of gene expression profiles, Jönsson and collaborators were able to categorise melanomas into four biologically relevant subgroups; MITF‐low/proliferative, high‐immune response, MITF‐high/pigmentation and normal‐like 19. Importantly, the MITF‐low/proliferative subtype, characterised by an absence of the expression of immune‐response genes, had only *BRAF/NRAS*‐mutated samples and more tumours with *CDKN2A* deletions, and was significantly associated with a poorer prognosis. Classification of primary melanomas by gene expression also resulted in these four classes, which could be collapsed into two classes associated with clinical outcome 62. The multi‐institutional TCGA (The Cancer Genome Atlas) initiative subsequently identified three transcriptomic subclasses, an immune group, a keratin group, and a MITF‐low group 4, 63. A subsequent analysis showed that these classifications comprised very similar biological entities (TCGA immune ∼ Lund high‐immune, TCGA keratin ∼ Lund normal‐like and MITF‐high/pigmentation, TCGA MITF‐low ∼ Lund MITF‐low/proliferative) 63.

#### Molecular markers of response/resistance to targeted therapy and immune‐checkpoint inhibition

The development of approved targeted therapies for patients with metastatic and early‐stage melanomas has been remarkable and driven by significant discoveries around the molecular mechanisms of melanomagenesis. Combined treatment with *BRAF* and *MEK* inhibitors achieves radiological responses in ∼70% of patients with *BRAF* ^V600^ mutations 68. A proportion of patients are intrinsically resistant to *BRAF* inhibitors, and most patients who initially respond will eventually exhibit resistance. The need to maximise the long‐term clinical benefit of this strategy remains a key challenge and molecular profiling may play a particularly important role in deciphering the mechanisms of response and resistance to targeted therapy.

One of the most frequently reported mutations leading to intrinsic *BRAF* resistance is loss of the phosphatase and tensin homolog (*PTEN*) gene. Decreased responses to *BRAF* inhibition in patients with *PTEN* loss is thought to be attributed to constitutive activation of the PI3K/AKT pathway which leads to cell proliferation and survival 69. *MITF* has also been shown to be an important regulator of response, and high *MITF* levels allow melanoma cells to evade cell death triggered by BRAF and MEK inhibitors 70, 71. Intriguingly, it has been shown that very low levels of *MITF* when co‐existing with high levels of receptor tyrosine kinase AXL (MITF^low^/AXL^high^ phenotype) represent a de‐dedifferentiated cellular state that displays innate resistance to BRAF inhibitors and increased invasiveness 20. A number of other mechanisms of intrinsic resistance have been suggested, reviewed in 72. The most common mechanism of acquired resistance is *via* reactivation of the MAPK/ERK pathway 72. Recently, studies have reported non‐mutational mechanisms for the acquisition of resistance through phenotype switching 21, 73, 74.

Immune checkpoint inhibitor (ICI) therapy has revolutionised melanoma therapy and resulted in unprecedented rates of long‐term disease control and survival in patients with metastatic disease. It is hypothesised that the mutational status of a cancer influences anti‐tumour immune and ICI responses, presumably by virtue of enhanced neoantigen formation due to increased number of non‐synonymous single‐nucleotide variants 75, 76. This phenomenon probably reflects an increased likelihood of forming neoantigens that will elicit T‐cell reactivity. Consistent with this notion, tumours with microsatellite instability resulting from acquired deficiency of DNA mismatch repair are also associated with enhanced response to PD‐1 blockade 77, 78. This has formed the basis for the first site‐agnostic drug approval made by the FDA, for anti‐PD1 therapy 79, 80. Studies *ex vivo* strongly support the dominance of mutational neoantigens as the targets for lymphocyte recognition of a tumour, and neoantigen expression and HLA binding characteristics have been shown to be surrogates for treatment response 75, 76, 81, 82. In keeping with this, mutational and neoantigen load have also recently been linked with clinical benefit from adoptive T cell immunotherapy 83. Further evidence suggests that clonal neoantigens may be particularly relevant 84. While genomic instability may feasibly provide sufficient genomic variation to promote an effective immune response, the mechanism relating DNA damage and genomic instability to ICI response is not fully understood and mutational load does not sufficiently explain all cases 85. Significant genomic heterogeneity between tumours can contribute to heterogenous clinical responses and this may account for some of the conflicting results seen in separate cohorts 86, 87.

Immune activation gene‐expression signatures have been shown to define distinct CM subtypes 19 and the prevalence of pre‐existing tumour infiltrating T cells has been shown to correlate with clinical response to anti‐PD1 immunotherapy 88. Although previous reports have suggested that the expression of cytolytic markers might correlate with response to anti‐CTLA4 76, these are based on small retrospective analyses and there has yet to be any specific gene expression signature that has been independently validated in this context. It is increasingly appreciated that the relationship of the tumour's mutational profile to immune dynamics is moderated by additional factors that affect expression, processing and immunogenicity of putative neoantigens. Accordingly, predictive approaches are now being paired with additional filters as well as expression data to evaluate somatic mutations which are adequately expressed and processed.

### Desmoplastic melanoma (DM): a CM subtype with an elevated mutational load

DM is a variant of CM, consisting of intradermal proliferations of spindled melanocytes, commonly associated with lymphoid aggregates, and typically found on chronically sun‐damaged skin of older individuals (Figure 1). The term DM initially referred to the association of invasive tumour cells with abundant stromal collagen, and therefore DM can be classified as pure and mixed, based on the degree of desmoplasia 89. Pure DMs have less frequent lymph node involvement and tend to display a less aggressive clinical course than mixed DM. DMs rank among the most heavily mutated types of cancer, with a mutation rate on average four‐fold higher than CMs, of which the great majority are attributed to UV mutagenesis 13. DMs also tend to have lower DNA copy number alterations than other melanoma subtypes; the few focal deletions that have been observed target *CDKN2A* and *NF1*, whereas amplifications affect *EGFR*, *CDK4*, *MDM2*, *TERT*, *MAP3K1*, *MET*, *YAP1* and *NFKBIE* 13. The promoter of *NFKBIE* has been identified as a recurrently mutated locus in 15–33% of samples 3, 13. This gene, coding for IkBϵ, inhibits downstream nuclear factor kappa B (NFκB) signalling by sequestering NFκB transcription factors in the cytoplasm (Figure 2) 13. Although also mutated in CM, promoter mutations are enriched in DM 3. No melanoma hotspot mutations in *BRAF* or *NRAS* have been identified in studies focusing on DM 13, 90, 91; the MAPK pathway seems instead to be activated by other mutations 13 (Table 1). Indeed, possible oncogenic MAPK mutations in this subtype of melanoma include alterations detected in *NF1*, *CBL*, *ERBB2*, *MAP2K1* and *MAP3K1*, as well as mutations that are hotspot in other types of cancers such as *BRAF* G469E, G466E and D594N and *NRAS* Q61H 13.

Following the recognition that somatic non‐synonymous mutational load might be associated with improved immune checkpoint responses, Eroglu and colleagues hypothesised that patients with DM may respond well to ICI therapies 92. In a retrospective analysis of pathology reports from 1058 patients with advanced melanoma treated with anti–PD‐1 or anti–PD‐L1 antibodies, Eroglu *et al* identified 60 patients with advanced DM, who overall had a high response rate to PD‐1 blockade. Whole‐exome sequencing data from 17 patients revealed driver *NF1* mutations in 14/17 samples (82.4%) and enrichment of loss‐of‐function mutations in *TP53* and *ARID2*. However, these mutations were not associated with response to PD‐1 blockade. These findings suggest that, despite the dense fibrous stroma that had been expected to limit immune infiltration, PD‐1/PD‐L1 blockade may be effective in patients with DM, supporting further clinical investigation of immune checkpoint blockade in these patients.

Additional rarer categories of CMs that have distinctive histopathological and molecular features include spitzoid melanoma 93, melanoma arising from giant congenital naevus 94 and melanoma in childhood 95, not reviewed herein.

### AM: numerous copy number changes and low point mutation burden

AM is a rarer histological variant arising on the palms, soles and nail beds and accounts for a greater proportion of melanomas in patients of African, Asian and Latin American descent 96, 97, 98, 99. When compared to CM tumours, AMs have a much lower single nucleotide mutational burden yet display a higher number of somatic structural aberrations 3, 44. The few AM samples sequenced to date demonstrate a lower contribution of the UV signature 3, 44. A handful of cases from subungual sites, however, do demonstrate a significant proportion of UV‐associated mutations, which might suggest that skin in these locations might not be completely protected from sun‐induced UV‐radiation 100.

A large proportion of AMs fall into the TWT subtype, with only 42–55% of tumours having mutations in *BRAF*, *NRAS* or *NF1* 3, 44 (Table 1). *KIT* mutation and amplifications are also AM drivers, with between 3 and 36% of tumours bearing these alterations 44, 52. A fraction of AMs also carry activating mutations in the promoter of *TERT* (between 9 and 41% of patients depending on the study 44, 46) and *TERT* gene amplifications are currently the only recognised adverse molecular prognostic indicators 101. *TERT* inhibition has been shown to be cytotoxic for AM cells *in vitro* 44 and, following both *in vivo* and *in vitro* evidence, *TERT* inhibitors are currently being proposed for clinical use 102. Interestingly, although *TERT* deregulation in UV‐exposed melanomas is caused by point mutations, about 45% of AMs have *TERT* copy number gains 3. *TERT* copy number may predict the outcome of high‐dose (HD)‐IFNα‐2b treatment in AM 103.

Genes frequently targeted by amplifications are *KIT*, *TERT*, *PAK1*, *CDK4* and *CCND1*, and genes recurrently deleted include *CDKN2A*, *PTEN* and *NF1* 44, 104 (Table 1). A study of 514 primary AM samples showed that the overall frequency of at least one aberration in *CDK4*, *CCND1* or *P16* ^*INK4a*^ was 82.7%. In this study, AM cell lines and patient‐derived xenografts containing cyclin dependent kinase 4 (CDK4) pathway aberrations were sensitive to CDK4/6 inhibitors 105 and clinical studies are anticipated (NCT03454919). There are other, infrequently altered genes identified by AM sequencing studies; for example, mutations of *MAP2K2* and loss of *ARID2* 44. Another subset of AMs show, like CMs, *MITF* amplifications 3. Interestingly, very few point mutations have been described in *TP53*, *PTEN*, *RAC1* or *RB1*, with these genes instead being targeted via amplifications or deletions.

Although AMs harbouring *BRAF* or *KIT* mutations may respond to the appropriate inhibitors, the majority of patients do not currently have any genotype‐specific treatment options. In light of the lower somatic mutation burden, it might be thought that the efficacy of ICIs may be lower in this subtype 106. However, small retrospective series have so far demonstrated that response rates are comparable to those in CM 107, 108. It remains to be seen whether the association of mutational and neoantigen load to ICI response is also observed in this subtype.

### UM: a sparsely mutated melanoma subtype with poor responses to modern systemic therapies

#### The genomic landscape of UM

UM has one of the lowest observed mutational densities across all tumours, estimated to be about 1.1 somatic mutations per Mb 4. Indeed, the burden of coding somatic mutations is comparable to that of paediatric cancers such as medulloblastoma and neuroblastoma 48. Nonetheless, a small number of recurrent somatic mutations have been observed. Activating mutations in the guanine‐nucleotide proteins *GNAQ* and *GNA11* occur in the great majority of tumours (a combined frequency of ∼85–92.5%), and in *CYSLTR2* (4%) and *PLCB4* (2.5%), all in a mutually exclusive manner 4, 56, as these may all activate the MAPK pathway 56, 109 (Figure 2, Table 1). Other significantly mutated genes in UM are *BAP1*, *EIF1AX* and *SF3B1*, which also form a second mutually‐exclusive subgroup 56 (Table 2). In addition to providing an insight into key molecular signalling and progression pathways, UM driver genes also associate with molecular subclasses and bear important prognostic implications (Table 2). Different studies have found a number of mutational signatures in these tumours, most notably one associated with ageing and explained by spontaneous deamination of 5‐methylcytosine, and others related to defects in nucleotide excision and in DNA mismatch repair 4, 48. There are currently no known drugs that target GNAQ/GNA11 and alternative pathway inhibitors have so far not shown clinical benefit in early phase trials, reviewed in 110. A number of trials are ongoing, targeting a range of UM signalling cascades 111. The clinical responses to ICIs have similarly been disappointing 112.

**Table 2:** Uveal melanoma driver genes and their prognostic significance

Gene	Gene function	Mutation frequency (%)	Association with metastases	Association with survival
*GNAQ*	Mediating signalling between G‐protein‐coupled receptors and downstream effectors and upregulated MAPK pathway	43–57	Similar frequencies reported between metastatic and non‐metastatic lesions	Mutations have not been linked to patient outcome 113
*GNA11*	Mediating signalling between G‐protein‐coupled receptors and downstream effectors and upregulated MAPK pathway	41–49	Present in 18/30 (60%) of UM metastases	Disease‐specific survival in *GNA11*‐mutant patients was 60 months, overall survival 50.6 months (from date of primary tumour), significantly poorer than those tumours lacking *GNA11* mutations 117
*BAP1*	Involved in tumour suppression, DNA damage response and proliferation	70–83	Inactivating somatic mutations in 26/31 (84%) of metastasising tumours. Also associated with Class 2 GEP, M3 and 8q gain.	Overall survival in BAP1 positive nuclear staining by IHC was 9.97 months (95% confidence interval 8.05–11.9) versus BAP1 negative by IHC 4.74 (3.49–6.0) 49, 118, 119
*EIF1AX*	Eukaryotic translation initiation factor	8–21	Mutant cases are associated with very low risk of metastases (only 2/28 cases)	*EIF1AX* mutant cases had a longer disease‐free survival than *EIF1AX* non‐mutant cases (190.1 vs. 100.2 months; *p* < 0.001) 113, 120, 121
*SF3B1*	Required for pre‐mRNA splicing	10–24	Intermediate risk of metastases – late‐onset (>5 years) metastases can occur	Although an association of mutated *SF3B1* with favourable prognosis was observed in the first few years 122, with longer follow up time, *SF3B1* mutant patients developed more metastases and tumours with D3 and *SF3B1* mutation showed a significant worse prognosis compared to wild‐type tumours 121, 123

Chromosomal copy number gains and losses (copy number alterations [CNAs]) are more common in UM, and the largest genomic studies have focused on these aberrations. Unsupervised hierarchical clustering of UM genomes based on CNAs reveals two main chromosomal subsets 4, 48, 113. Aberrations in chromosome 3 are the main distinguishing feature between the two main subsets and TCGA refers to these subsets as Disomy 3 (D3) and Monosomy 3 (M3) 49. It has long been recognised that M3 is associated with poor prognosis and high metastatic risk, while tumours with D3 correlate with good prognosis and rarely lead to disseminated disease 114. The metastatic rate for tumours with M3 ranges from 0 to 48% 115, and the M3 genotype has been shown to be superior to clinicopathologic factors as a prognostic indicator 114. Gain of the long arm of chromosome 8q is also associated with poor prognosis 116. The M3 cluster is characterised by aberrations in *BAP1*, as well as 8q gain, but the extent and type of this chromosomal gain varies between the two sub‐clusters. The gain of 8q, where *MYC* is located, does not contain this oncogene and *MYC* transcript levels do not correlate with 8q status 4, 48, so perhaps this gain is targeting another genomic region. The D3 cluster further subdivides into two subsets, one characterised by little aneuploidy, gains of chromosome 6p (short‐arm) and somatic mutations in *EIF1AX*, and the second with gains of chromosomes 6p and 8q (long‐arm) and somatic mutations in *SF3B1*. Given the prevalence of observed alterations, it has been proposed that mutations in *GNAQ*, *GNA11, CYSLTR2* or *PLCB4* represent an early event, followed by loss of chromosome 3 and mutation of *BAP1* in the case of M3, and by mutation of *EIF1AX* or *SF3B1* in the case of D3 48. The TCGA study also clustered samples using transcriptional and methylation profiles, which largely aligned with the original CNA clusters 4.

#### Gene expression profiling in UM

UMs can also be stratified according to the GEP classification described earlier, and into the same prognostically relevant molecular classes 33 and this has become the standard of care for molecular testing in a number of oncology centres 124. Recently, Class 1 tumours have been subdivided into two subgroups, Class 1A (2% of patients 5‐year metastatic risk) and Class 1B (21% of patients 5‐year metastatic risk) 125, based on the differential expression of *CDH1* and *RAB31*. Class 1A tumours are also associated with D3 and *EIF1AX* mutations. Class 2 UM tumours exhibit a dedifferentiated stem‐cell‐like and epithelioid phenotype that is associated with M3 and *BAP1* mutations and confers a high metastatic risk 118, 125. They can be subclustered into Class 2A and 2B, where Class 2B cases harbour a loss of chromosome 8p that makes them even more aggressive with an earlier onset of metastases relative to Class 2A 126. Unlike CM, multiple groups have shown that the prognostic accuracy of GEP outperforms clinicopathologic features and chromosomal gains and losses in predicting metastases 127, 128, 129.

### MM: a rare and aggressive subtype

MMs are rare and have a particularly aggressive clinical course 130, 131. Similar to AM, MM is characterised by a higher number of chromosomal structural aberrations and a lower mutational burden than CM 3, 54. Mutations in *BRAF*, *NRAS* or *NF1* in MM are less prevalent than in CM, with loss of *PTEN* (4–25% of samples 3, 54) mutation or amplification of *KIT* (7–25% of MM samples 3, 54, 132) and *CCND1* or *CDK4* 104 being more common (Table 1). In fact, Hayward and colleagues identified a previously unappreciated set of driver genes shared between UM and MM, with two‐thirds of TWT MM showing activating mutations in *GNAQ* and *SF3B1*. Additionally, some studies 52 suggest that losses of *CDKN2A* are more common in AM and MM than in CM, though estimates vary 3, 44, 50.

Targeted therapies against mutation of *KIT* have failed to show convincing therapeutic efficacy in MMs 133. The immune checkpoint blocking antibodies have shown variable efficacy in phase II and retrospective studies 134, 135.

## Conclusion

Despite significant progress in the understanding of CM biology, our ability to assess the likelihood of recurrence and death for any individual patient remains conspicuously limited. Assessment of an individual CM patient's risk is currently based on the AJCC recommendations, which consider traditional staging factors such as Breslow thickness and ulceration 136. However, over two‐thirds of CM‐related deaths occur in patients diagnosed with stage I or II disease 137, and, as the incidence of melanoma continues to increase, the absolute number of such ‘low risk’ patients who ultimately relapse and die is rising 138. National guidelines do not currently recommend intensive surveillance and adjuvant therapy for stage I‐IIA disease 139. Additional strategies for prognostication in this early‐stage CM cohort, particularly those with biological propensity to metastasise and who might benefit from modern survival‐prolonging adjuvant therapies 140, 141, 142, would clearly be beneficial. However it seems clear that while there is tremendous enthusiasm to integrate molecular biomarkers into clinical practice, no such markers or signatures fulfil the necessary criteria for inclusion into the AJCC melanoma staging or as a component of any validated clinical tool 136. More studies are also needed to determine which type of specimen and approach yields the highest success rate. The creation of large, prospective, multi‐institution registry studies that harness the power of electronic data sharing should improve on some of the shortcomings of current prognostic tools including; relatively small study populations, short follow‐up and lack of internal and external validation. These studies will be needed to address this unmet clinical need for patient stratification in CM.

Distinct melanoma subtypes harbour somatic aberrations on the same key pathways, but the affected genes may be different (Table 1). Accordingly, it is evident that the current genomic classification of melanomas devised by TCGA may work well with CM and with DM to some extent, but a similar classification that informs therapeutic options is needed for AM and MM, where more than 50% of tumours may fall into the TWT subtype.

Why melanomas from non‐CSD regions have such different genomic landscapes to those from CSD tissues remains an open question. Apart from the fact that cells from these different regions have varying exposure to UV‐induced mutagenesis, another possible explanation may lie in the different lineages from which these melanocytes originate, and the different microenvironments they inhabit. Therefore, the question remains, if the CM driver mutations arose in melanocytes from glabrous skin, and *vice versa*, would melanocytes transform and form tumours? Or, are the melanocyte lineages sufficiently different that different mutations are needed to progress to malignancy? Would the microenvironment play a significant role in melanocyte transformation? *In vitro* experiments with cell lines from different melanocytic lineages and *in vivo* experiments in model organisms such as mice and zebrafish should help address this fundamental question.

Clearly, although good progress has been achieved for patients with CM, therapeutic options and response remain poor in patients with other melanoma subtypes. Recent studies exploring prognostic markers and potential therapeutic targets are helping bridge this gap, and as more genomes from rarer melanoma subtypes are sequenced, our understanding of targeted therapy and response should improve. Most of these tumoural genomes originate from patients of European descent, and a further important question is whether findings in these populations can be translated to patients from other ethnicities – particularly in AM and MM which constitute a higher proportion of melanoma cases in non‐European descent populations.

## Author contributions statement

RR described the prognostic and therapeutic implications of molecular aberrations across the melanoma subtypes, and drafted sections of the introduction and conclusion. CDRE and CMA described the genomic landscape of melanoma subtypes, as well as sections of the introduction and conclusion. PF provided the H&E images and caption, and commented on the histopathologic aspects of the manuscript. DJA and CDRE provided overall supervision and leadership, as well as comments and suggestions across the entire manuscript. All authors approved the final version.
